# Some Remarks at an Eye Clinic

**Published:** 1882-04-20

**Authors:** A. G. Hobbs

**Affiliations:** Professor of Diseases of the Eye, Ear and Throat, in Southern Medical College, Atlanta, Georgia


### Southern Medical Record:

EDITORS:

T. S. Powell, M.D. W. T. Goldsmith, M.D. R. C. Word, M.D.

*R. C. WORD, M.D., Managing Editor.*

☞All Communications and Letters on Business connected with the Record must be addressed to the Managing Editor.

Vol. XII. ATLANTA, GA., APRIL 20, 1882. No. 4.

### ORIGINAL AND SELECTED ARTICLES.

### SOME REMARKS AT AN EYE CLINIC.

BY A. G. HOBBS, M. D.,

Professor of Diseases of the Eye, Ear and Throat, in Southern Medical College, Atlanta, Georgia.

Reported by J. C. Beauchamp, M. D.

January 2d, 1882, John Sammonds, male, æt. 26 years; occupation, a farmer. History: Nineteen years ago had sore eyes, which seemed to be rather intractable, and was not cured. We have now in this case trichiasis, entropion, and as a result of this condition of things, pannus. This has produced considerable softening of the cornea, and bulging of its coats.

This trouble was brought about by the long continuance of the conjunctivitis, resulting in hypertrophy of the lid, from infiltration and cell proliferation of the connective tissue. The lid as well as the eye-lashes became inverted, and by continual rubbing over the cornea produced pannus. This man can only see to read print as large as the heading of the Constitution. Now the practical question is, how shall we relieve our patient? The indications are to restore the lid and eye-lashes to their proper position. By so doing, we remove the source of irritation to the cornea, and the pannus will then get well of itself. The operations for the relief of this condition, practiced up to a recent date, have not proved very satisfactory in their results. The operation usually resorted to for the relief of trichiasis has been epilation of the hairs. This, while it may relieve the pannus, leaves the patient with an unsightly baldness. For the relief of the entropion, it has been recommended to dissect a crescent-shaped or wedge-shaped piece of skin from the upper lid, and bring it together with sutures.

This method of operating does not result, generally, in a permanent cure. I shall perform an operation on this man, first suggested by Dr. Hotz, of Chicago, in 1880. I assisted at the first operation of this kind performed in New York. Have made the operation myself only six times.

It consists in removing an eliptical piece of the intigument of the upper lid and dissecting out the orbicularis muscle down to the upper border of the tarsal cartilege. Now you will see by its bluish color that I have reached the cartilege—be careful here, gentlemen, in the use of your knife, because the conjunctiva is very close, and is easily cut through.

I shall now bring the edges of the wound together with fine black silk sutures, but in doing so you will notice that I take a middle stich. Now watch where the needle enters as I take this middle stitch—for here lies the point in the new operation—it dips down and passes through the upper border of the tarsal cartilege before it pierces the integument on the opposite side. You will see as I draw the knot that the edges of the skin are pulled tightly down on to the cartilege. This tarsal cartilege is the purchase by which the contracting cicatrix turns the edges of the lid and the lashes back to their normal position, (I might almost say *abnormal* position, in this man's case, since the lashes have been turned under the greater part of his life.)

You will see I have made four sutures. We will order cold water dressings to keep down any undue inflammatory reaction, and remove the threads on the fourth day.

As the hour is up, we will make the operation on the other eye on next Thursday.

P. S.—At the present writing, April 3, the patient's lids and lashes are in a perfectly natural position; scar scarcely perceptible, and the pannus is nearly all cleared away. He reads ordinary print at eight inches, and, with a minus glass, sight is good for reading at eighteen inches. His myopia is due to the elongation of the eye-ball, from the protrusion of the cornea.

